# Downregulation of Wnt3 Suppresses Colorectal Cancer Development Through Inhibiting Cell Proliferation and Migration

**DOI:** 10.3389/fphar.2019.01110

**Published:** 2019-10-01

**Authors:** Xiaobo Nie, Fulin Xia, Ying Liu, Yun Zhou, Wenling Ye, Panha Hean, Jiming Meng, Huiyang Liu, Lei Liu, Jianxun Wen, Xuequn Ren, Wei-Dong Chen, Yan-Dong Wang

**Affiliations:** ^1^Key Laboratory of Receptors-Mediated Gene Regulation and Drug Discovery, School of Medicine, Henan University, Kaifeng, China; ^2^Department of General Surgery, Affiliated Huaihe Hospital of Henan University, Kaifeng, China; ^3^Key Laboratory of Molecular Pathology, School of Basic Medical Science, Inner Mongolia Medical University, Hohhot, China; ^4^State Key Laboratory of Chemical Resource Engineering, College of Life Science and Technology, Beijing University of Chemical Technology, Beijing, China

**Keywords:** Wnt3, colorectal cancer, HCT-116, proliferation, apoptosis, glycolysis

## Abstract

The aberrant expression of Wnt3 has linked to several types of human malignancies. However, it is not known for its role in tumorigenesis of colorectal cancer (CRC). Herein, we show that Wnt3 is upregulated in human CRC tissues and is essential for the CRC progression. Knockdown of Wnt3 in human CRC cells delayed tumor formation in nude mouse xenografts through silencing of canonical Wnt pathway and glycolysis. We further found that silencing of Wnt3 enhanced the sensitivity of CRC cells to cisplatin through inducing apoptotic cell death. Taken together, it demonstrates that Wnt3 is a novel clinical biomarker for the detection of CRC and plays an important role in colorectal tumorigenesis. Therefore, downregulation of Wnt3 will be a valuable strategy in CRC treatment.

## Introduction

Colorectal cancer (CRC) is the third most common cancer and the fourth leading cause of cancer-related death worldwide, with more than 1,400,000 new cases diagnosed annually (Navarro et al., 2017). The occurrence of this polygenetic disease is mainly ascribed to the interactions between genetic predisposition and environmental factors; among them, the most frequent risk factor is the Western pattern diet (Bultman, 2017). In early stages, CRC is curable through enterectomy combined with chemoradiotheraphy, and the 5-year survival rate could exceed 90%, whereas the rate will decline to be < 30% when it progresses to middle or advanced stage. The routine uses of magnetic resonance imaging and computed tomography scanning could clearly determine the number, size, location, and the boundary of the tumor, whereas it can be subject to the relative higher expense in developing regions and missed diagnosis for smaller, shallow lesions and tumors whose scanning density is close to benign. With the advantages of safe for operation and clear observation of local mucosa, colonoscopic examination cannot only be used for early diagnosis of CRC but also for the most effective treatment of stage I CRC (Holme et al., 2014). However, it also limited by proficient operating skill and situations when patients have symptoms of intestinal obstruction or with invasion and metastasis tumors (Chittleborough et al., 2018). Therefore, it is still meaningful to explore effective and highly accurate biomarkers and detailed mechanisms contributing to improved diagnostic and therapeutic management of CRC.

Named for *Drosophila* wingless (wg) and mouse homolog Int-1 (Wnt-1), Wnt genes are highly conserved during species evolution and encode for 19 glycoproteins in humans. The central role of Wnt signaling is to control embryonic development as well as tissue homeostasis (Majidinia et al., 2018; Steinhart and Angers, 2018). The canonical Wnt pathway is activated upon binding of secreted Wnt ligands to the corresponding receptor frizzled family (FZD) and coreceptor low-density lipoprotein receptor related protein (LRP5/6). Then, the dishevelled (DVL) is phosphorylated and begins to form a polymer that can inactivate the destruction complex through recruiting AXIN and GSK3β; thereby, β-catenin is avoiding being phosphorylated and accumulates in nucleus and forms complexes with coregulators and coactivators of transcription factors, thus activating downstream genes such as cyclin D1 and c-Myc, which ultimately brings about changes of cellular activities including cell proliferation, motility, and polarity (Clevers and Nusse, 2012). Numerous studies have proved that mutations or abnormal expression of components in Wnt pathways are closely related to the development of human malignancies (Anastas and Moon, 2013). Notably, aberrant activation of Wnt signaling is frequently observed in human CRC, and a comprehensive genome-scale analysis found that 93% CRC patients carrying loss-of-function mutations in 16 different genes of the Wnt signaling pathway such as biallelic inactivation of the negative regulator adenomatous polyposis coli or activating mutations of β-catenin (Cancer Genome Atlas Network, 2012). Fortunately, many exciting therapeutic agents targeting Wnt signaling pathway have been developed for CRC treatment despite certain challenges in drug discovery (Novellasdemunt et al., 2015). However, the roles and detailed mechanisms of Wnt pathway in the progression of cancers remain obscure due to the abundant number of the Wnt family members and their specific roles in different types of tumors. Therefore, extensive research efforts should be made at targeting Wnt pathway in cancer therapy.

Wnt3 belongs to Wnt family and has been proven to be relevant in lung, gastric, hepatic, colorectal, and breast cancer tumorigenesis (Nambotin et al., 2012; Nakashima et al., 2012; Wu et al., 2012; Voloshanenko et al., 2013; Wang et al., 2016). To explore the role of Wnt3 in tumorigenesis of CRC, we examined Wnt3 expression levels in human CRC tissues and paired normal colorectal tissues, as well as the effects of dysregulation of Wnt3 on CRC cellular activities and tumor formation in nude mice. Our results indicated that Wnt3 is highly expressed in CRC tissues and corresponding cell lines and plays a tumorigenic role in CRC development as well as contributes to the drug-resistant phenotype of CRC cells. Furthermore, knockdown of Wnt3 in CRC cells suppresses CRC cell growth, migration, and tumor growth and induces the apoptosis and drug sensitivity of CRC cells. These results suggest that Wnt3 plays an oncogenic role in colorectal carcinogenesis and could be a promising clinical diagnostic biomarker and therapeutic candidate for CRC.

## Materials and Methods

### Immunohistochemistry

The paraffin-embedded tissue microarray for CRC was purchased from Shanghai Outdo Biotech (Shanghai, China). The immunohistochemistry was performed as described. Briefly, the tissues were incubated at 65°C for 1 h and rehydrated through an ethanol gradient. The section was boiled in 1 mM ethylenediaminetetraacetic acid buffer (pH 7.5) for 30 min for antigen retrieval and quenched by immersing in 3% hydrogen peroxide. After blocking the nonspecific binding with 5% sheep serum albumin for 30 min, the section was incubated with a rabbit anti-Wnt3 antibody (Abcam, UK) (1:500 dilution in 5% bovine serum albumin in distilled water) overnight at 4°C, then rinsed three times with *phosphate*-*buffered* saline (PBS) and incubated for 30 min at 37°C with a drop of secondary antibody. After washing with PBS three times, the section was visualized with 3′3-diaminobenzidine tetra-hydrochloride, counterstained with hematoxylin. All sections were observed under a microscope, and images were randomly taken with the same exposure time and light intensity.

### Collection of Colorectal Tissue Specimens

The study was approved by the Ethics Committee of Affiliated Huaihe Hospital of Henan University, Kaifeng, and written informed consent was obtained from all patients. Human colorectal samples were collected from the Department of General Surgery during surgery on patients with CRC, who underwent complete surgical resection for the disease at Affiliated Huaihe Hospital between February 2016 and January 2017. The patients were selected based on pathological diagnosis of CRC without any other malignancies or histories of preoperative anticancer treatments. The tissue samples were immediately snap frozen and stored in liquid nitrogen until subsequent analysis.

### Cell Culture

The cell line of HCT-116 was purchased from School of Basic Medicine of Peking Union Medical College (Beijing, China). Cells were cultured in Dulbecco’s modified Eagle’s medium (Thermo, USA) supplemented with 10% fetal bovine serum (FBS) (Thermo, USA) and antibiotics (100 U/ml penicillin and 0.1 mg/ml streptomycin) (Gibco, USA) at 37°C in a humidified atmosphere of 5% CO_2_.

### Selection of Stable CRC Cells That Overexpress or Knockdown Wnt3

The pGPU6 plasmid that express short hairpin RNA (shRNA) targeting *Wnt3* messenger RNA (mRNA) and pEX4 plasmid that overexpress *Wnt3* were constructed (GenePharma, China) and amplified. HCT-116 cells were seeded into a six-well plate at a density of 4 × 10^5^ cells per well 24 h before transfection. Cells were transfected with 3 μg of Wnt3-shRNA (NC-shRNA) or Wnt3-pEX4 (NC-pEX4) using 7.5 μl Lipofectamine 2000 (Invitrogen, USA) in serum- and antibiotic-free medium. The medium was aspirated 24 h later and replaced with fresh medium containing Geneticin (G418) at the final concentration of 750 μg/ml. Fresh medium with G418 was replaced every other day for the selection of transduced cells. Ten days later, single cell with green fluorescence was selectively transferred to wells of a 96-well plate using flow cytometer and expanded. The Wnt3-shRNA clones showing the most efficient knockdown or Wnt3-pEX4 clones showing the most efficient overexpression as compared to the control group were identified by quantitative real-time PCR (qRT-PCR) analysis and used for further analysis.

### RNA Extraction and qRT-PCR

Total RNA was extracted from frozen tissue samples or selected cell clones using an RNA simple Total RNA kit (Tiangen, China) according to the manufacturer’s instructions. The RNA was quantified using a nanodrop reader, and 2 μg of RNA was reverse transcribed to complementary DNA (cDNA) using the Strand cDNA Synthesis kit (Thermo, USA). Briefly, total RNA was mixed with 1 μl oligo-dT primer, made up to a 12-μl mixture with nuclease-free water, and heated at 65°C for 5 min, followed by chilling on ice. After primer hybridization, 8 μl reaction volume containing 5× reaction buffer, RiboLock RNase inhibitor (20 U/μl), 10 mM deoxynucleotide mix, and RevertAid M-MuLV reverse transcriptase (200 U/μl) were added to the RNA and incubated for 60 min at 42°C followed by 5 min at 95°C. The qRT-PCR analyses were performed using 50 ng cDNA per reaction with SYBR green assay with primer set designed to amplify target genes with the following conditions: denaturation at 95°C for 3 min followed by 40 cycles of denaturation at 95°C for 30 s and annealing and extension at 62°C for 40 s. Melting curve analysis (65–95°C) was routinely performed to confirm the specificity of primers at the end of the assay. The relative mRNA expression levels of targeted genes were normalized against the β-actin using the comparative ΔΔCt method, and relative fold change of gene was calculated by the equation 2^−ΔΔCt^. Sequences of the primers used for real-time PCR are given in **Table 1**.

**Table 1:** The sequences of the primers used in qRT-PCR.

Primer name	Sequences
h-actin-F	ACTGGGACGACATGGAGAAA
h-actin-R	CTGGATAGCAACGTACATGG
h-Wnt3-F	CCACAACACGAGGACGGAGA
h-Wnt3-R	CGCCCAGCCACACACTTC
h-FZD7-F	CGCTCATGAACAAGTTCGGC
h-FZD7-R	CATGAGAAGGGGAAGGCGG
h-CTNNB1-F	GCGCCATTTTAAGCCTCTCG
h-CTNNB1-R	AAATACCCTCAGGGGAACAGG
h-CCND1-F	AGTTGCAAAGTCCTGGAGCC
h-CCND1-R	GTTTCCACTTCGCAGCACAG
h-c-Myc-F	GTCAAGAGGCGAACACACAAC
h-c-Myc-R	TTGGACGGACAGGATGTATGC
h-GluT1-F	TCTGGCATCAACGCTGTCTTC
h-GluT1-R	CGATACCGGAGCCAATGGT
h-HK2-F	GAGCCACCACTCACCCTACT
h-HK2-R	CCAGGCATTCGGCAATGTG
h-LDHA-F	TTGACCTACGTGGCTTGGAAG
h-LDHA-R	GGTAACGGAATCGGGCTGAAT
h-PKM2-F	ATGTCGAAGCCCCATAGTGAA
h-PKM2-R	TGGGTGGTGAATCAATGTCCA

### Protein Extraction and Immunoblot

Frozen tissue or cell samples were lysed by radioimmunoprecipitation assay buffer containing 1% NP-40, 0.5% deoxycholate, 0.1% sodium dodecyl sulfate (SDS) (Beyotime, China) in the presence of protease inhibitor and centrifuged at 15,000×*g* for 10 min at 4°C. The supernatant fraction was collected, and the protein concentration was determined by bicinchoninic acid assay (Beyotime, China). Equal amounts of protein extract (30 μg per well) were separated by 10% SDS poly acrylamide gel electrophoresis. Separated proteins were electrotransferred to a 0.2-μm polyvinylidene fluoride transfer membrane and blocked in 5% nonfat milk in 1× Tris-buffered saline, 0.1% Tween 20 for 1 h. The blot was incubated with antibodies at 4°C overnight followed by horseradish-peroxidase-conjugated secondary antibody (Proteintech, China). Antibodies recognizing Wnt3 and cyclin D1 was used at a dilution of 1:2,000 (Abcam, UK), antibodies recognizing poly ADP ribose polymerase (PARP), cleaved PARP, caspase 3, cleaved caspase 3 (Proteintech, China), phospho-β-catenin (Thr41/Ser45), and β-catenin (Cell signaling, USA) were used at a dilution of 1:1,000. Antibody against β-actin (Proteintech, China) was used as loading control for the experiment. The membrane was then incubated with SuperSignal West Pico Chemiluminent Substrates (Thermo, USA) for 2 min and developed under automatic multifunction chemiluminescent detection system (Tanon, China). The signal was quantified by densitometry using Image-J Software.

### Cell Proliferation Assays

Cell proliferation ability was determined using Real-Time Cellular Analysis (RTCA, ACEA Biosciences, USA). In brief, cells were seeded into E-plate 16 and incubated in 5% CO_2_ at 37°C. The proliferation index was recorded every 15 min to 54 h using RTCA software, and the cellular growth index was generated from the average values of six wells for each group.

### Cell Cycle and Cell Apoptosis Analysis

For cell cycle assay, HCT-116 cells that stably knockdown Wnt3 were seeded into 60-mm plates and incubated without FBS for 12 h and with FBS for another 36 h. Subsequently, cells were harvested and suspended with ice-cold 70% ethanol and kept at 4°C for 4 h, then incubated with 20 μg/ml propidium iodide (PI) at room temperature for another 30 min. Cell cycle status was assessed with a NovoCyte^TM^ flow cytometer (ACEA, USA) equipped with NovoExpress^TM^ software according to the manufacturer’s recommendations.

Annexin V-FITC/PI apoptosis kit (Multi Sciences, China) was used to detect cellular apoptosis. Briefly, HCT-116 cells that stably overexpress or knockdown Wnt3 were seeded in six-well plates and incubated for 12 h, and then, cells were incubated with cisplatin (8 μg/ml) for another 36 h. Before analysis, cells were harvested and suspended with 500 μl binding buffer. Subsequently, another 5 μl Annexin-V-FITC and 10 μl PI were added in sequence and incubated at room temperature for 5 min; cell apoptosis was analyzed using NovoCyte^TM^ flow cytometer, and data analysis was performed using NovoExpress^TM^ software.

For apoptosis-related genes analysis, HCT-116 cells that stably overexpress or knockdown Wnt3 were seeded in six-well plates and incubated for 12 hours, and then cells, were incubated with 12 μg/ml cisplatin for another 24 h (pEX4 groups) or 48 h (shRNA groups). Cells were harvested to analyze the transcriptional levels of proapoptotic genes including *BAX*, cytochrome C (C*YCS*), caspase 3 (*CASP3*), *CDKN1B* (p27), *FAS*, and *TP53* (p53).

### Cell Migration Assays

Equal numbers of HCT-116 cells that stably knockdown Wnt3 were seeded into six-well plates, followed by scraping with a 100-μl sterile pipette tip to generate two linear regions devoid of cells completely. The plates were then washed three times with PBS and refreshed with 2% FBS medium. Cells were photographed and incubated in a 5% CO_2_ at 37 °C. The cellular migrated distance was monitored, and images were taken at 24 and 48 h under a microscope.

Moreover, the cell migration rate was also determined by RTCA. In brief, cells were seeded into the upper chamber of CIM-Plates 16 without FBS, and the lower chamber was added with complete growth medium, and then, the device with CIM-Plates 16 was incubated in 5% CO_2_ at 37°C. The migration index was recorded every 30 min to 72 h using RTCA software. The cellular migration index was generated from the average values of four wells for each group.

### Colony Formation Assay

HCT-116 cells that stably overexpress or knockdown Wnt3 were counted, and a total of 500 cells/well were seeded evenly into six-well plates and incubated at 37°C for 2 weeks. Cells were washed three times with PBS, fixed with 75% ethanol, and stained with crystal violet solution for 1 h and photographed. Colony number was automatically counted by Image Pro Plus 6.0 (IPP 6.0) software.

### Cisplatin Sensitivity Assay

To determine the effect of Wnt3 knockdown in CRC cells on the sensitivity for cisplatin, HCT-116 cells that stably knockdown Wnt3 or the control cells were seeded in 96-well plates at a density of 8 × 10^3^ cells/well and incubated for 12 h. Then, cells were treated with 4 or 8 μg/ml cisplatin or treated with PBS as control. Twenty-four hours later, cells were incubated with 10 μl 3-(4,5-dimethylthiazol-2-yl)-2,5-diphenyltetrazolium bromide (5 mg/ml) for 4 h, and then, medium was aspirated to change into 100 μl dimethyl sulfoxide. The absorbance at 450 nm was measured using an EnSpire^TM^ microplate reader (PerkinElmer, USA), and the cellular viability was calculated by the ratio of optical density values of cells treated with 4 or 8 μg/ml to nontreated group. Meanwhile, cells seeded in 6-cm plate with the same treatment were used for immunoblotting.

### Glucose Uptake and Lactate Production Assays

HCT-116 cells that stably overexpress or knockdown Wnt3 and their controls were seeded in 12-well plates at a density of 4 × 10^5^ cells/well and incubated with high-glucose DEME for 48 h, the medium without cell was used as control for the experiment. The culture medium was collected and centrifuged at 300×*g* for 10 min at room temperature to remove the residual cells, and then, the supernatant fraction was collected for use. The glucose levels were determined using a glucose colorimetric assay kit (BioVision, USA), and lactate levels were measured using a lactate colorimetric assay kit (BioVision, USA) according to the manufacturer’s instructions.

### Xenograft Tumor Assay in Nude Mice

BALB/c nude mice were purchased from Beijing Vital River Laboratories (Beijing, China) and maintained in a specific pathogen-free environment under a standard 12:12-h light/dark cycle. Five-week-old mice were fed standard rodent chow and injected subcutaneously in the left and right flanks with HCT-116 cells that stably overexpress (3.5 × 10^6^) or knockdown Wnt3 (5 × 10^6^) and their controls in 200 μl of serum-free PBS. The mice were killed 24 days later, and tumors were excised and weighted. Tumors were homogenized and lysed for further RT-PCR analysis. This study was carried out in accordance with the recommendations of the National Institutes of Health guidelines for the care and use of laboratory animals. The protocol was approved by the Animal Research Ethics Committee of School of Medicine of Henan University.

## Statistical Analysis

Statistical analysis and figures were carried out using the GraphPad Prism. One-way ANOVA analysis or two-sided Student’s *t* test was performed to evaluate statistical significance. Kaplan–Meier survival analysis was used to evaluate the prognosis of patients with CRC. The correlation between Wnt3 expressions and clinicopathological parameters was assessed by the Pearson correlation analysis. Error bar for the experiments represents the standard deviation of the mean value (mean value ± SD) from triplicate experiments. *P* values lower than 0.05 were considered as statistically significant. One asterisk, two asterisks, and three asterisks represent *P* < 0.05, *P* < 0.01, and *P* < 0.001, respectively

## Results

### Wnt3 Was Overexpressed in CRC

**Figure 1:**
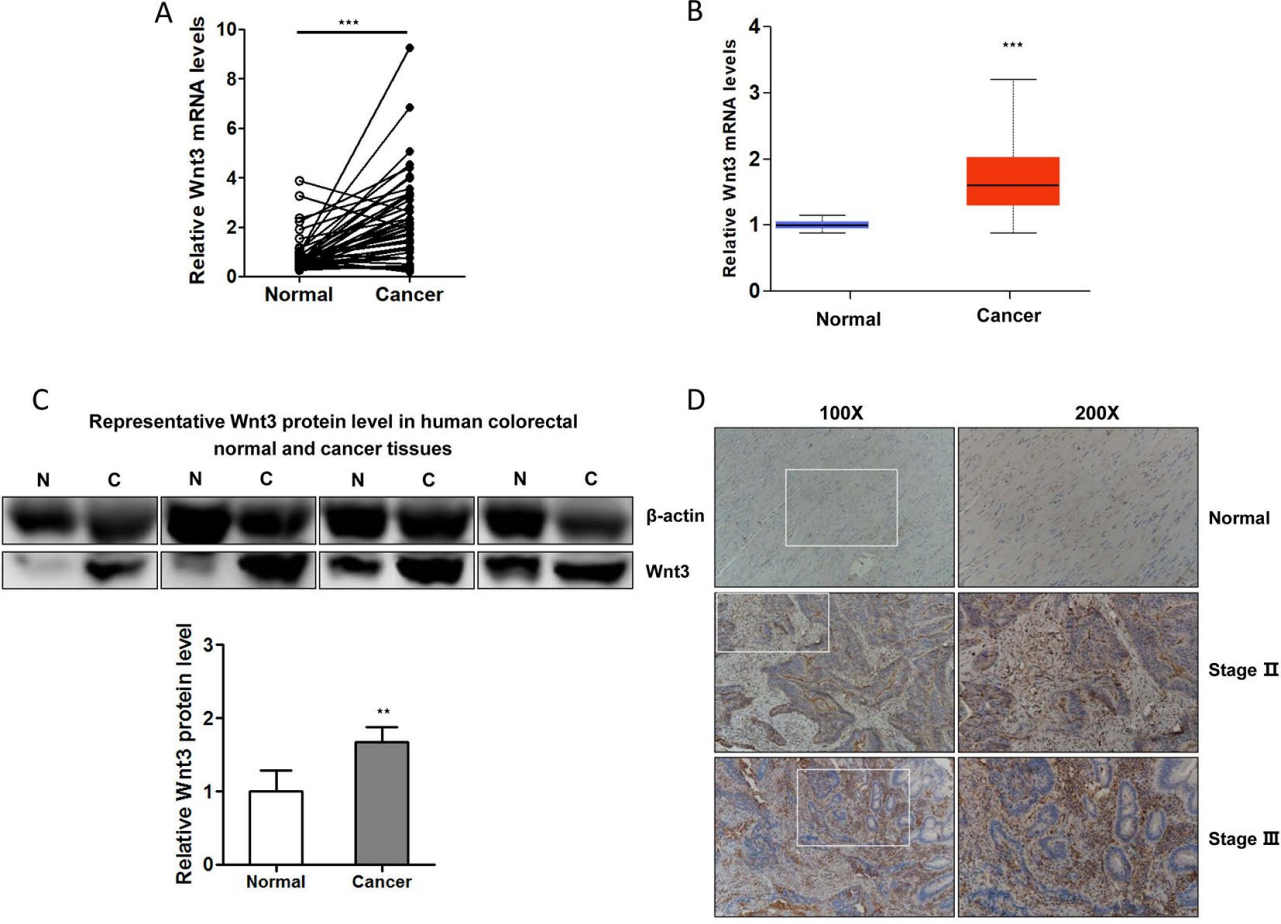
Expression of Wnt3 is upregulated in human colorectal cancer (CRC) tissues. **(A)** *Wnt3* messenger RNA (mRNA) levels in CRC tumor tissues and paired normal tissues were examined by quantitative real-time PCR (qRT-PCR; *N* = 46, ***P < 0.001). **(B)** Online TCGA data analysis of *Wnt3* mRNA level in colorectal normal and cancerous tissues from CRC patients (*N* = 279). **(C)** Representative immunoblot of Wnt3 protein expression in human CRC tissues and corresponding nontumor ones by Western blot analysis, normalized to β-actin protein levels. Densitometry was used to quantify relative Wnt3 protein levels normalized to β-actin (**P < 0.01). **(D)** Representative immunohistochemical staining of Wnt3 in human normal or CRC tissue sections (100× and 200×).

Several studies have revealed that Wnt3 plays an important role in cellular proliferation and tumorigenesis. To investigate whether Wnt3 is aberrantly activated in CRC microenvironment, we examined the Wnt3 transcript levels in 46 pairs of CRC and matched non-neoplastic colorectal samples by qRT-PCR analysis. The transcript level of Wnt3 gene was found to be overexpressed significantly in CRC tissues compared with normal tissues (**Figure 1A**). We also validated the result through analyzing data from the Cancer Genome Atlas (TCGA) (**Figure 1B**). In addition, immunoblot detection demonstrated a similar upregulation of Wnt3 protein in CRC tissues than that in corresponding nontumor ones (**Figure 1C**). In a further effort to validate whether Wnt3 expression is elevated in the CRC tissues, we performed immunohistochemistry in tissue microarray containing 30 pairs of matched CRC specimens. Representative sections of Wnt3 expression in tissues with different pathological grades are shown in **Figure 1D**.

### Correlation Between Wnt3 Expression and Clinicopathological Parameters

**Figure 2:**
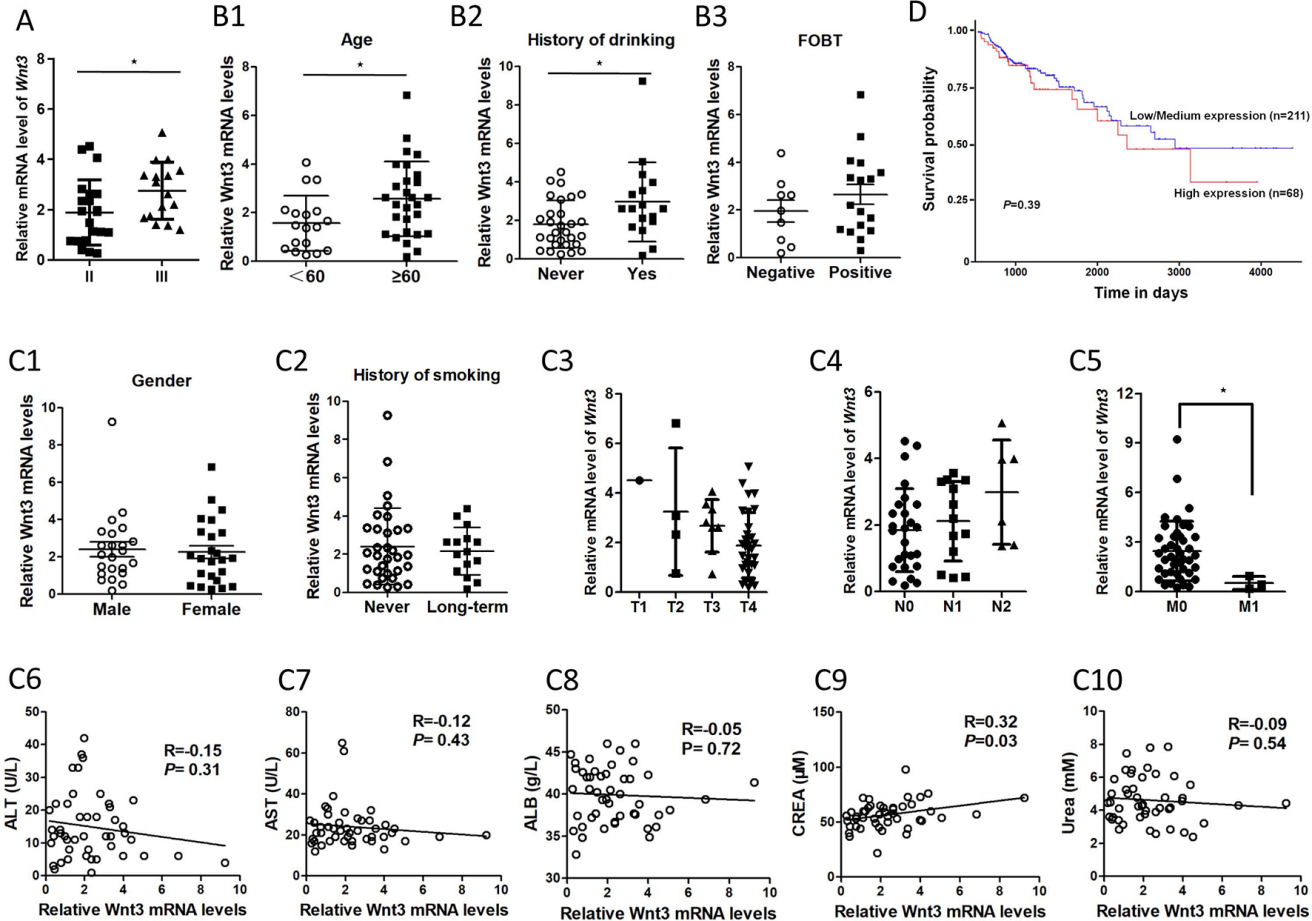
The association between *Wnt3* messenger RNA (mRNA) levels and various clinicopathological factors of colorectal cancer (CRC) patients. **(A)** *Wnt3* mRNA levels of CRC tumor tissues in stage II and III. (*P < 0.05). **(B)** *Wnt3* mRNA levels in CRC patients with different age, drinking history, and fecal occult blood test (FOBT) (*N* = 46, *P < 0.05). **(C1**–**C2)** *Wnt3* mRNA levels in CRC patients with different gender and smoking history (*N* = 46, *P<0.05). **(C3**–**C5)** *Wnt3* mRNA levels of CRC tumor tissues in TNM stages are shown (*P < 0.05). **(C6**–**C10)** The association between *Wnt3* mRNA levels and level of alanine transaminase (ALT), aspartate aminotransferase (AST), albumin (ALB), creatinine (CREA), and urea levels in CRC patients (*N* = 46). **(D)** The overall survival curve of CRC patients (*N* = 279) according to *Wnt3* mRNA level were analyzed with data from the TCGA.

The association between Wnt3 mRNA levels and clinicopathological features of CRC was further analyzed. The data demonstrated that the Wnt3 level was elevated gradually in stage II and III (**Figure 2A**). Advanced CRC often accompanies with multiple organ metastases; however, CRC tissues with metastases displayed low level of Wnt3 compared with nonmetastatic ones (**Figure 2C5**). Moreover, the expression of Wnt3 was found to be associated with age, history of drinking, and fecal occult blood test (FOBT) (**Figure 2B**). However, no significant correlation was observed between Wnt3 expression and variables such as T stage, lymph node, gender, smoking status, and level of ALT, AST, ALB, CREA, and urea (**Figure 2C**). Furthermore, we performed a bioinformatics investigation using the data obtained from TCGA database. A poor survival rate was observed in patients with high level of Wnt3 (**Figure 2D**), even though the result did not reach statistical significance. Taken together, these results suggest that Wnt3 is upregulated in CRC and perhaps exerts a carcinogenic effect in the occurrence and development of CRC.

### Overexpression of Wnt3 Promotes the CRC Cellular Growth and Tumor Growth

**Figure 3:**
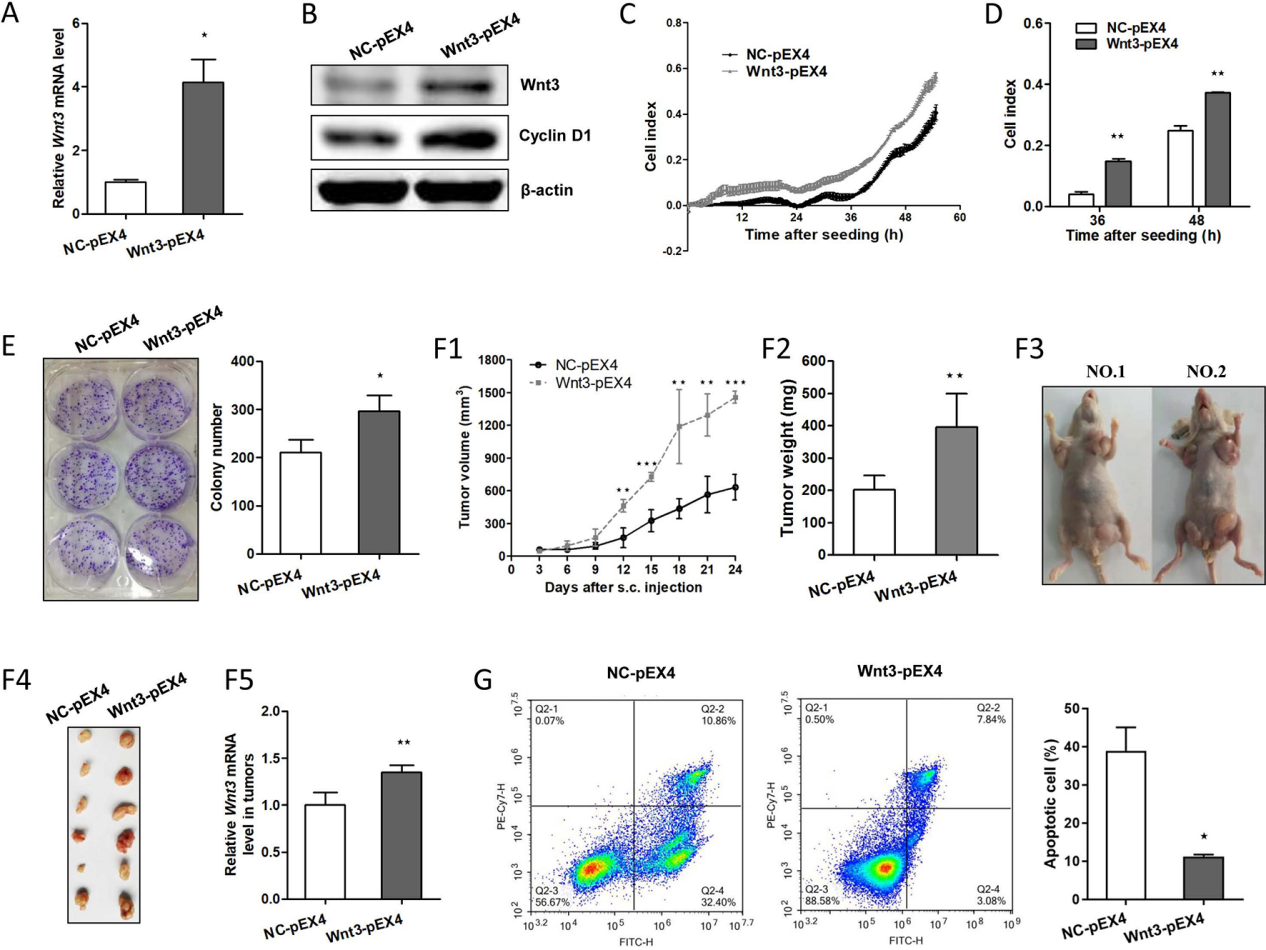
Overexpression of Wnt3 accelerates colorectal cancer (CRC) and tumor growth and inhibits cellular apoptosis induced by cisplatin. **(A)** Representative qRT-PCR analysis of *Wnt3* messenger RNA (mRNA) level in selected HCT-116 clone cells that stably overexpress Wnt3-pEX4 plasmid (Wnt3-pEX4) or control one (NC-pEX4). **(B)** Representative immunoblot showing Wnt3 and cyclin D1 protein levels extracts from Wnt3-pEX4 or NC-pEX4 HCT-116 cells. **(C** and **D)** Real-time cell analysis (RTCA) showed the cell proliferation ability was obviously accelerated in Wnt3-pEX4 cells, and the proliferation rate of Wnt3-pEX4 cells at the 36 and 48 h were analyzed. The proliferation index was recorded every 15 min to 54 h postseeding using RTCA software. **(E)** Three representative images of colony formation assay of NC-pEX4 or Wnt3-pEX4 cells, and colony number was automatically counted by IPP 6.0 software, the colony formation assays were performed in triplicate. **(F)** The real-time tumor size, tumor weight after killing, representative nude mice and dissected tumors, and Wnt3 mRNA level (*N* = 6) in tumor tissue lysates are shown. **(G)** NC-pEX4 and Wnt3-pEX4 cells were treated with 8 µg/ml of cisplatin for 36 h after seeding; cell apoptosis was analyzed by flow cytometry analysis. The cellular apoptotic rate was a sum of early and late apoptotic rates. *P < 0.05, **P < 0.01, ***P < 0.001 compared with the NC-pEX4 group.

To investigate the potential carcinogenic effect of Wnt3 on CRC cells, we constructed HCT-116 cells that could stably overexpress Wnt3 gene with G418 selection. As shown in **Figure 3A**, *Wnt3* mRNA levels were elevated obviously through Wnt3 plasmid transfection. Immunoblot analysis showed a similar increase in the Wnt3 protein levels as compared with the control groups (**Figure 3B**). To examine whether elevated Wnt3 could promote the CRC cellular growth, proliferation index of cells was monitored *via* RTCA system. Overexpression of Wnt3 significantly accelerated the proliferation and migration of HCT-116 cells (**Figures 3C, D**, **Supplementary Figure 1A**). Meanwhile, similar results were obtained from colony formation assays (**Figure 3E**). Furthermore, nude mice were subcutaneously injected with HCT-116 cells stably expressing exogenous Wnt3-pEX4 or NC-pEX4 plasmid into the right and left flanks, respectively. We found that overexpression of Wnt3 promoted the tumor growth obviously in nude mice (**Figure 3F**). We found that Wnt3 upregulation resulted in a significantly accelerated tumor growth. Collectively, these data demonstrated that the Wnt3 plays a tumor-promoting role in the development of CRC.

### Overexpression of Wnt3 Activates the Canonical Wnt Pathway and Induces the Drug Resistance to Chemotherapeutic Agents

To clarify the potential mechanism by which overexpression of Wnt3 promotes the development of CRC, we investigated the expression of genes in the canonical Wnt signaling pathway. Strikingly, the protein levels of target genes in canonical Wnt pathway such as cyclin D1 are elevated (**Figure 3B**); β-catenin is also activated due to a decrease ratio in phosphorylated β-catenin (p-β-catenin) to total β-catenin (t-β-catenin) in Wnt3-pEX4 cells compared to the control ones (**Supplementary Figure 2**), and the mRNA levels of *c-Myc* and *CCDN1* (encoding cyclin D1) genes in tumor tissues formed by Wnt3-pEX4 cells on nude mice are also elevated (**Supplementary Figure 3A**), giving rise to the changes of series of cellular activities including excess cellular proliferation and tumor growth *in vivo*. We further questioned whether elevated Wnt3 was involved in the resistance to chemotherapeutic agents to promote the progression of CRC. To test this assumption, we treated the same concentration of cisplatin with HCT-116 cells stably expressing Wnt3-pEX4 plasmid or control one, we were surprised to find that overexpression of Wnt3 significantly reduced the cellular apoptosis (**Figure 3G**). Simultaneously, the mRNA levels of proapoptotic genes such *as BAX, CASP3, CDKN1B*, *FAS*, and *TP53* were all downregulated in Wnt3-pEX4 cells after the treatment of cisplatin (**Supplementary Figure 4A**), indicating a decrease in drug sensitivity when Wnt3 is overexpressed in CRC cells.

### Inhibition of Wnt3 Suppresses CRC Cell Growth, Migration, and Tumor Growth

**Figure 4:**
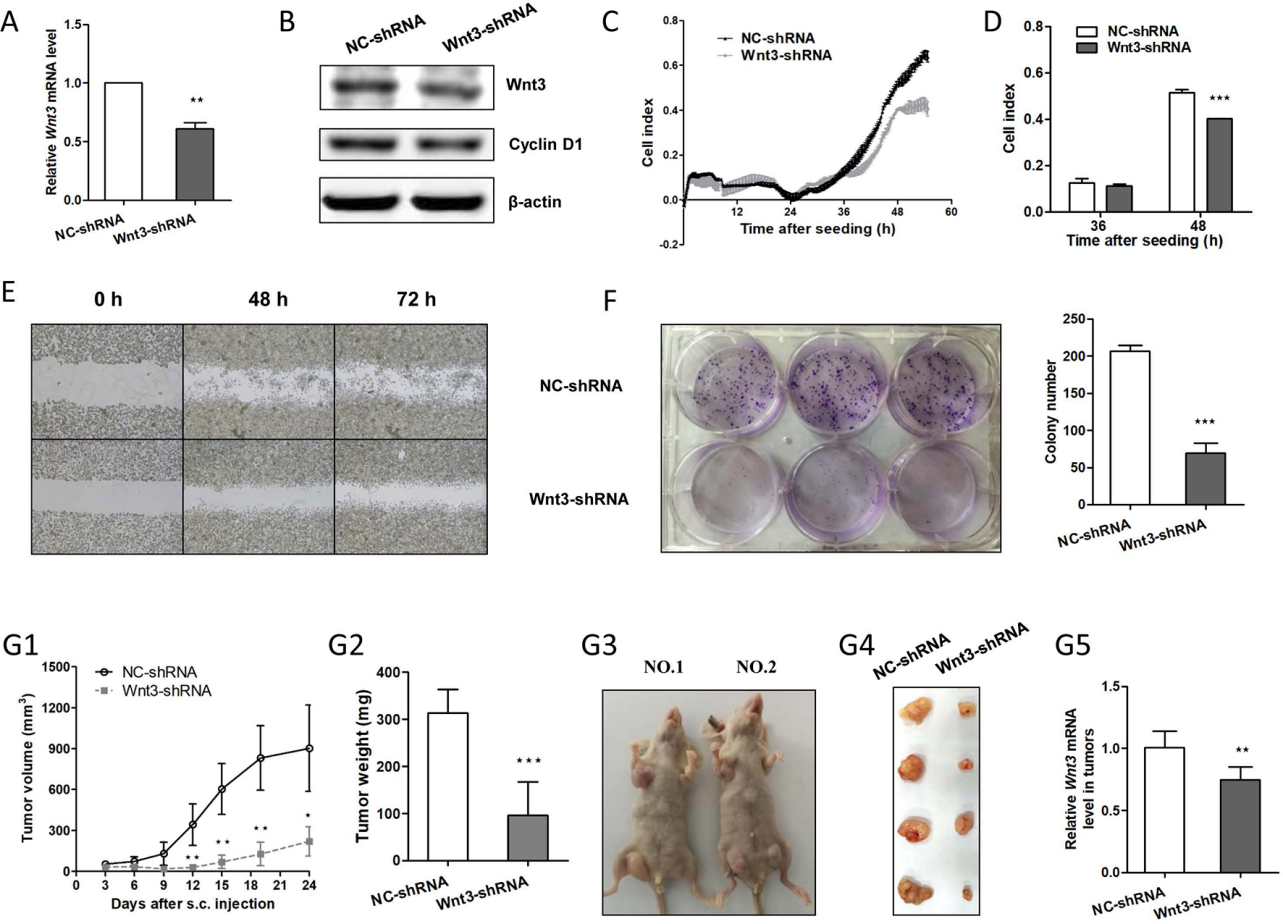
Knockdown of Wnt3 inhibits colorectal cancer (CRC) proliferation, migration, and tumor growth. **(A)** Representative quantitative real-time PCR (qRT-PCR) analysis of *Wnt3* mRNA level in selected HCT-116 clone cells that stably overexpress Wnt3-shRNA plasmid (Wnt3-shRNA) and control one (NC-shRNA). **(B)** Representative immunoblot showing Wnt3 and cyclin D1 protein levels extracts from Wnt3-shRNA and NC-shRNA cells. **(C** and **D)** Real-time cell analysis (RTCA) showed the cell proliferation ability was obviously inhibited in Wnt3-shRNA cells, and the proliferation rate of Wnt3-shRNA cells at the 36 and 48 h were analyzed. The proliferation index was recorded every 15 min to 54 h postseeding using RTCA software. **(E)** Representative photographs of Wnt3-shRNA or NC-shRNA cells taken at 0, 48, and 72 h postwound (×40). **(F)** Three representative images of colony formation assay of NC-shRNA or Wnt3-shRNA cells, and colony number was automatically counted by IPP 6.0 software; the colony formation assays were performed in triplicate. **(G)** The real-time tumor size, tumor weight after killing, representative nude mice and dissected tumors, and Wnt3 mRNA level (*N* = 6) in tumor tissue lysates are shown. *P < 0.05, **P < 0.01, ***P < 0.001 compared with the NC-shRNA group.

Since Wnt3 was identified as an oncogenic gene in CRC development as mentioned above, we next sought to investigate whether downregulation of Wnt3 could inhibit CRC tumorigenesis. We took advantage of the convenient shRNA technique and G418 selection to stably knockdown the endogenous Wnt3 mRNA in HCT-116 cells. As was expected, *Wnt3* mRNA and protein level were both suppressed, verified by qRT-PCR and immunoblot analysis, respectively (**Figures 4A, B**). In addition, a marked inhibition of cellular proliferation rate was detected in cells expressing Wnt3-shRNA (**Figures 4C, D**), indicating that knockdown Wnt3 inhibits cell proliferation. We further performed the colony formation assay, wound healing assay, and cell migration assay to test the effect on tumor formation and cellular migration ability. As shown in **Figures 4E, F** and **Supplementary Figure 1B**, silencing of Wnt3 led to the reduction in colony number and migration capacity. To further determine whether knockdown of Wnt3 regulates tumorigenesis, HCT-116 cells that stably knockdown Wnt3 or the control cells were injected subcutaneously. Knockdown of Wnt3 indeed suppressed CRC cell tumorigenicity (**Figure 4G**). Together, these data suggest that knockdown of Wnt3 inhibits CRC cell proliferation and invasiveness, both *in vitro* and *in vivo*.

### Silencing of Wnt3 Expression Arrests Cell Cycle and Induces Apoptosis in CRC Cells

**Figure 5:**
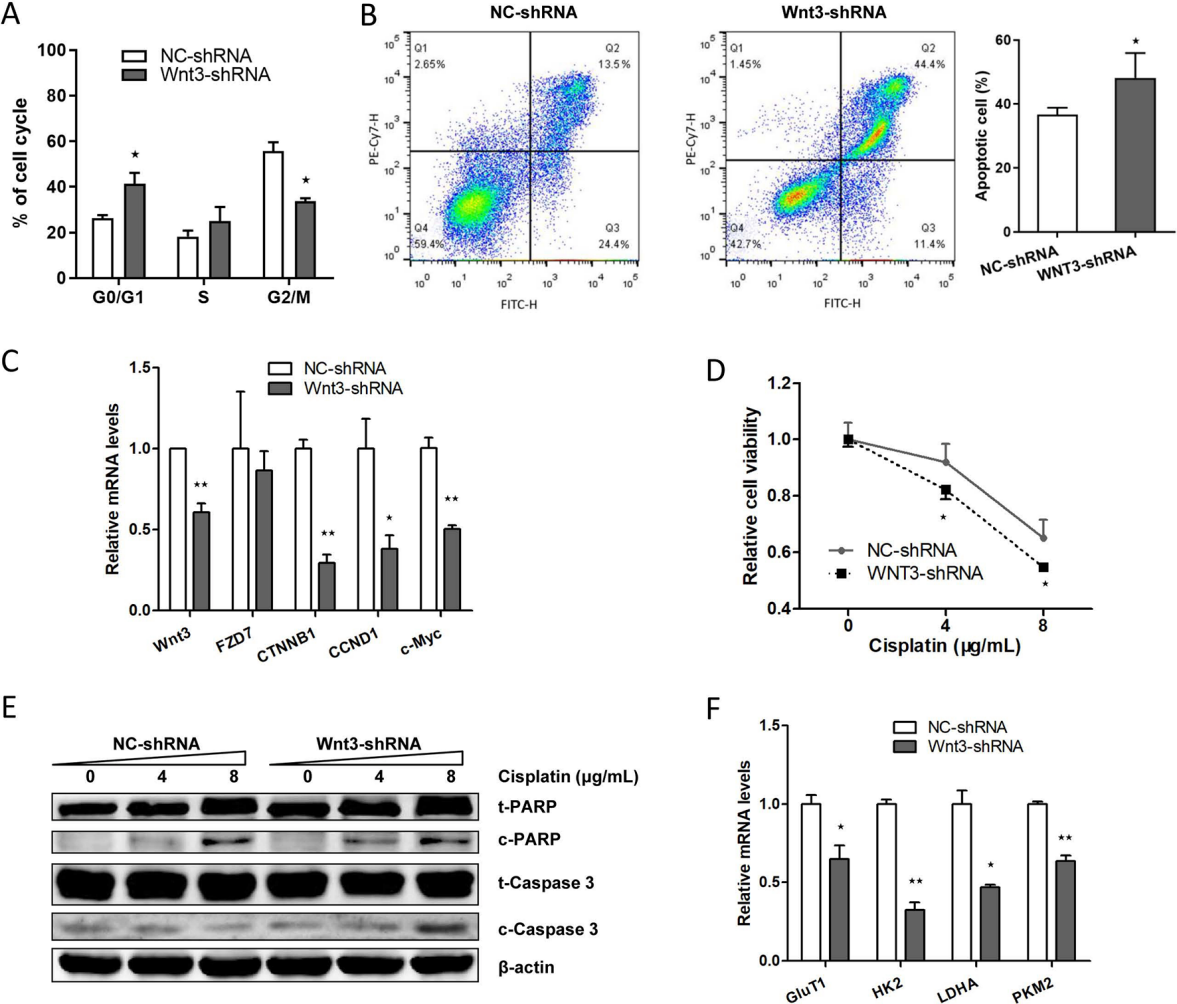
Downregulation of Wnt3 arrests the cell cycle, induces the apoptosis of CRC cells, inhibits the expression level of genes involved in canonical Wnt pathway and glycolysis. **(A)** Flow cytometry analysis of cell cycle distribution in Wnt3-shRNA and NC-shRNA cells. Cell cycle arrest was analyzed with a NovoCyte^TM^ flow cytometer and the results are representative of three independent experiments. **(B)** Wnt3-shRNA or NC-shRNA cells were treated with 8 µg/ml of cisplatin for 36 h after seeding, and the cell apoptosis was analyzed by flow cytometry analysis. The cellular apoptotic rate was a sum of early and late apoptotic rates. **(C)** Representative quantitative real-time PCR (qRT-PCR) analysis of mRNA levels of crucial genes involved in canonical Wnt pathway in NC-shRNA and Wnt3-shRNA cells. **(D)** Wnt3-shRNA and NC-shRNA cells were treated with the indicated concentration of cisplatin for 24 h, and the cell viability was assayed by 3-(4,5-dimethylthiazol-2-yl)-2,5-diphenyltetrazolium bromide. The data are presented as mean ± SD (*N* = 6). **(E)** Western blot analysis of PARP (t-PARP), cleaved PARP (c-PARP), caspase 3 (t-caspase 3), and cleaved caspase 3 (c-caspase 3) protein levels in lysates extracts from Wnt3-shRNA or NC-shRNA cells, normalized to β-actin protein levels. **(F)** Representative qRT-PCR analysis of mRNA levels of crucial genes involved in glycolysis in NC-shRNA and Wnt3-shRNA cells. *P < 0.05, **P < 0.01 compared with the NC-shRNA group

We proceeded to assess the effect of Wnt3 depletion on cell cycle and apoptosis in CRC cells. The flow cytometry was performed to assay the cell cycle distribution of HCT-116 cells stably expressing Wnt3-shRNA or NC-shRNA. The results demonstrated that a higher percentage of cells expressing Wnt3-shRNA were arrested in the G0/G1 phase (**Figure 5A**), while on the contrary, a much lower percentage of cells were in the G2/M phase, compared with NC-shRNA cells, indicating the inhibitory effect of Wnt3 silencing on cell cycle regulation. Next, we investigated the impact of Wnt3 depletion on the cell apoptosis of CRC cells that stably knockdown Wnt3 through treating cisplatin. The data illustrated that silencing of Wnt3 increased number of apoptotic cells relative to the control group (**Figure 5B**). Furthermore, the mRNA levels of some proapoptotic genes such as *CDKN1B* and *TP53* were upregulated in Wnt3-shRNA cells treated with cisplatin, compared with control ones (**Supplementary Figure 4B**). Taken together, these results reveal that silencing of Wnt3 expression arrests cell cycle and induces apoptosis in CRC cells.

### Downregulation of Wnt3 Inhibits the Canonical Wnt Pathway and Induces the Drug Sensitivity to Chemotherapeutic Agents

We further determined the impact of Wnt3 deficiency on the signal transduction of canonical Wnt signaling pathway. Silencing Wnt3 expression in CRC cells reduced the transcript levels of β-catenin (encoded by *CTNNB1*) and downstream genes including *c-Myc* and *CCND1* (**Figure 5C** and **Supplementary Figure 3B**) and led to an increased proportion of p-β-catenin (**Supplementary Figure 2**), revealing the mechanism of inhibited cellular proliferation and migration capacities and slower tumor growth *in vivo*. In contrast, the expression of Wnt coreceptors Frizzled 7 is not affected by the knockdown of Wnt3 (**Figure 5C**). We then treated cisplatin on CRC cells stably expressing Wnt3-shRNA or NC-shRNA. It was found that silencing of Wnt3 significantly inhibited the cellular viability of CRC cells in a dose-dependent manner, indicating an increase in drug sensitivity when Wnt3 was inhibited in CRC cells (**Figure 5D**). Moreover, Wnt3-knockdown HCT-116 cells exhibited elevated cleavage of PARP and caspase 3 compared with control cells (**Figure 5E**). Thus, Wnt3 depletion enhances cisplatin-induced caspase-dependent apoptosis.

### Silencing of Wnt3 Inhibits Glycolysis *via* c-Myc in Canonical Wnt Pathway

Given the fact that c-Myc is capable of regulating glycolysis pathway and simultaneously tempered by canonical Wnt signaling pathway, we sought to determine whether Wnt3 knockdown could inhibit the glycolysis *via* the c-Myc in CRC cells. Consistent with our prediction, knockdown of Wnt3 reduced the c-Myc target genes involved in the glycolysis pathway, including *GLUT1*, *HK2*, *LDHA*, and *PKM2* (**Figure 5F**). Consistently, the mRNA levels of these genes were all downregulated in tumor tissues formed by Wnt3-shRNA cells on nude mice (**Supplementary Figure 3B**). While on the contrary, mRNA levels of *HK2* and *LDHA* genes were elevated in tumor tissues formed by Wnt3-pEX4 cells (**Supplementary Figure 3A**). To further determine the effect of Wnt3 on the glycolysis, we measured the glucose uptake and lactate production in HCT-116 cells with overexpression or knockdown of Wnt3. The data showed that overexpression of Wnt3 did not lead to an obvious increase in glucose uptake and lactate production in HCT-116 cells. On the contrary, knockdown of Wnt3 did not inhibited lactate production, whereas it did decrease glucose uptake (**Supplementary Figure 5**), indicating that silencing of Wnt3 perhaps inhibits glycolysis in CRC cells.

## Discussion

Wnt signaling is one of highly conserved pathways during species evolution, and it is critical for embryonic development and tissue homeostasis (Rijsewijk et al., 1987). Secretory Wnts are a family of 19 glyproteins that play crucial roles in the normal signal transduction of Wnt pathway; altered activities of Wnt proteins or deregulation of Wnt levels can result to the disease etiology. In recent decades, a large amount of studies have reported the close relationship between the deregulation of Wnt signaling pathway and a variety of human diseases, including hereditary diseases such as familial metaphyseal dysplasia and polycystic kidney disease (Goggolidou and Wilson, 2016; Kiper et al., 2016), neurodegenerative disease as well as metabolic diseases (Ackers and Malgor, 2018; Folke et al., 2018), several types of human malignancies including breast cancer, gastric cancer, chronic lymphocytic leukemia, and CRC (Wu et al., 2012; Poppova et al., 2016; Wang et al., 2016). However, complete knowledge of the expression pattern of Wnt3 in CRC tissues and cell lines and molecular mechanism of its role in CRC tumorigenesis are undefined yet, although Voloshanenko and colleagues have documented an elevation of Wnt3 in colorectal tumors (Voloshanenko et al., 2013).

In the present study, we elucidated the clinical significance of Wnt3 in human CRC. Consistent with the bioinformatics analysis using the data obtained from TCGA database, we found that Wnt3 was overexpressed in CRC tissues, both in transcriptional and translational levels compared with paired control ones. In addition, high Wnt3 expression level is correlated with tumor grade, age, drink status, and FOBT results. More importantly, a lower survival rate was related with a higher expression of Wnt3, even though it is not significant in statistics. Other Wnt family members including Wnt1, Wnt2, and Wnt3a were also found to be elevated in CRC tissues (Qi et al., 2014; Kramer et al., 2017; Zhang et al., 2018). These data revealed that expression levels of Wnt3 could serve as a valuable diagnostic biomarker for CRC patients.

In the status of tumor microenvironment, the activation of canonical Wnt signaling pathway inhibits the phosphorylation of β-catenin and leads to the accumulation of β-catenin in the nucleus, which activates the downstream genes such as cyclin D1 and c-Myc and give rise to the malignant transformation and excessive proliferation (Zhan et al., 2017). However, no research has reported the tumorigenesis effect of Wnt3 in CRC. According to our observation, overexpression of Wnt3 in CRC cell line accelerated the cellular proliferation and tumor growth in nude mice, through activating the β-catenin-dependent target oncogenes like c-Myc and cyclin D1 in Wnt signaling. It is verified that Wnt3 plays a tumorigenic role in colorectal carcinogenesis. In the following study, we addressed the question whether Wnt3 could serve as a potential therapeutic target for the treatment of CRC. In order to explore this hypothesis, we knocked down Wnt3 in HCT-116 cells and selected stable clones. The data demonstrated that downregulation of Wnt3 could inhibit cellular proliferation and colony formation due to cell cycle arrest in G1/S phase, suppress cellular migration ability, and inhibit tumor growth *in vivo*, through antagonizing the canonical Wnt signaling pathway.

It has been recognized that aerobic glycolysis is the main metabolic way adopted by most cancer cells, and c-Myc has the ability to promote the glycolysis through transcriptionally activating its target genes correlated in the aerobic glycolysis pathway (He et al., 2015; Sheng and Tang, 2016). We observed that Wnt3 deficiency inhibited glycolysis through suppressing the glucose uptake and the expression of crucial genes controlling cellular glycolytic rate in cancer cells, such as *GLUT1*, *HK2*, *LDHA*, and *PKM2*. We speculated that alteration of these genes in Wnt3-deficient cells is partially due to the silencing of c-Myc. In the treatment of CRC, therapeutic efficacy has been notably challenged by resistance to conventional chemotherapy drugs, such as cisplatin, which lead to the high mortality and development of recurrences or distant metastases (Galluzzi et al., 2014). In our study, it is found that Wnt3 depletion also increased the cellular apoptosis *via* upregulating the expression of some proapoptotic genes, activating caspase 3 cleavages and an increase in PARP, enhanced the sensitivity of HCT-116 cells to cisplatin by facilitating caspase-dependent apoptosis, which is opposite to the result that overexpression of Wnt3 suppresses the apoptosis of CRC cells in the treatment of cisplatin. Therefore, it is suggested that downregulation of Wnt3 may represent a promising strategy for the treatment of human CRC.

In summary, the above findings have uncovered that the expression level of Wnt3 was upregulated in CRC cancerous tissues. When Wnt3 is overexpressed, it activates the canonical Wnt pathway, thus leading to the accelerated growth of CRC cells and tumors. Conversely, suppression of endogenous Wnt3 in CRC cells inhibited cellular proliferation, migration, and tumor growth and promoted the apoptosis under cisplatin, through inhibiting the canonical Wnt pathways and glycolysis. Downregulation of Wnt3 may represent a promising strategy for the treatment of human CRC.

## Data Availability Statement

The datasets generated for this study are available on request to the corresponding author.

## Ethics Statement

This study was carried out in accordance with the recommendations of National Institutes of Health guidelines for the care and use of laboratory animals. The protocol was approved by the Animal Research Ethics Committee of School of Medicine of Henan University.

## Author Contributions

XN, W-DC, and Y-DW contributed conception and design of the study; XN, FX, YL, YZ, WY, PH, JM, HL, LL, JW, and XR performed the experiments; XN wrote the first draft of the manuscript; W-DC and Y-DW wrote sections of the manuscript. All authors contributed to manuscript revision, read and approved the submitted version.

## Funding

This work is supported by the National Natural Science Foundation of China (Grant Nos. 81472232 and 81970726 and Grant No. 81270522), Program for Science & Technology Innovation Talents in Universities of Henan Province (HASTIT, Grant No. 13HASTIT024), and Plan for Scientific Innovation Talent of Henan Province to W-DC; Henan Provincial Natural Science Foundation (Grant No. 162300410034), the National Natural Science Foundation of China (Grant No. 81700731), and the Scientific Research Fund of Henan University (Grant No. 2015YBZR051) to XN; the National Natural Science Foundation of China (Grant No. 81672433, No. 81970551 and No. 81370537), the Fundamental Research Funds for the Central Universities and Research Projects on Biomedical Transformation of China-Japan Friendship Hospital (Grant No. PYBZ1803), and the Fundamental Research Funds for the Central Universities (Grant Nos. PYBZ1706 and XK1802-8) to Y-DW.

## Conflict of Interest

The authors declare that the research was conducted in the absence of any commercial or financial relationships that could be construed as a potential conflict of interest.

## Abbreviations

ALB, albumin; ALT, alanine transaminase; AST, aspartate aminotransferase; CRC, colorectal cancer; CREA, creatinine; DVL, dishevelled; FOBT, fecal occult blood test; FZD, frizzled; LRP, LDL receptor related protein; qRT-PCR, quantitative real-time PCR; SDS-PAGE, SDS polyacrylamide gel electrophoresis.
