# Supplementary material for: Downregulation of Wnt3 Suppresses Colorectal Cancer Development Through Inhibiting Cell Proliferation and Migration
Source: Front Pharmacol. 2019 Oct 1;10:1110. doi: 10.3389/fphar.2019.01110 (PMC6779829; doi:10.3389/fphar.2019.01110)
Supplement: Supplementary file 1 [file Presentation_1.pptx]

## Slide 1
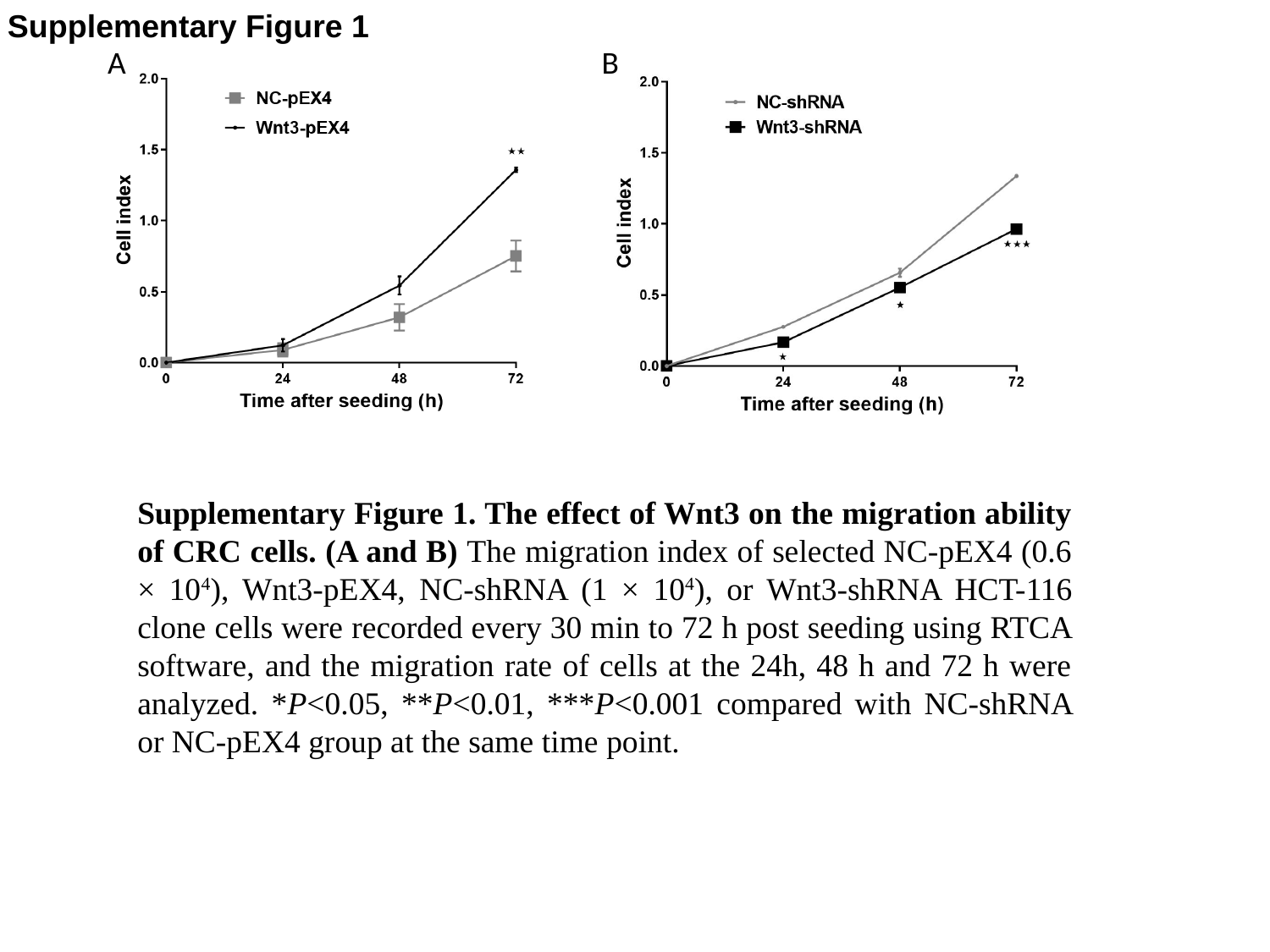

Supplementary Figure 1
A
B
Supplementary Figure 1. The effect of Wnt3 on the migration ability of CRC cells. (A and B) The migration index of selected NC-pEX4 (0.6 × 10^4^), Wnt3-pEX4, NC-shRNA (1 × 10^4^), or Wnt3-shRNA HCT-116 clone cells were recorded every 30 min to 72 h post seeding using RTCA software, and the migration rate of cells at the 24h, 48 h and 72 h were analyzed. *P<0.05, **P<0.01, ***P<0.001 compared with NC-shRNA or NC-pEX4 group at the same time point.

## Slide 2
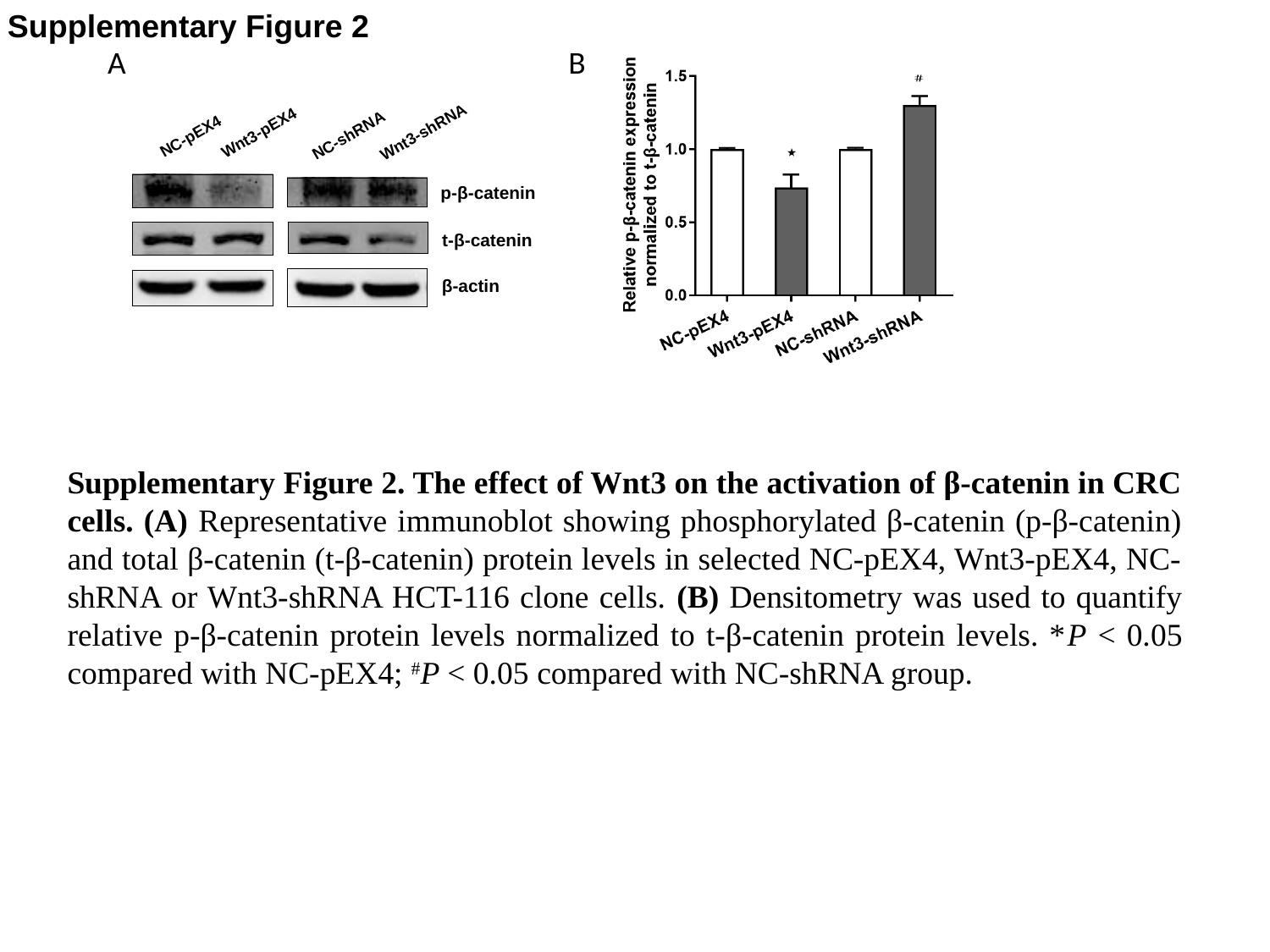

Supplementary Figure 2
A
B
Wnt3-pEX4
Wnt3-shRNA
NC-shRNA
NC-pEX4
p-β-catenin
t-β-catenin
β-actin
Supplementary Figure 2. The effect of Wnt3 on the activation of β-catenin in CRC cells. (A) Representative immunoblot showing phosphorylated β-catenin (p-β-catenin) and total β-catenin (t-β-catenin) protein levels in selected NC-pEX4, Wnt3-pEX4, NC-shRNA or Wnt3-shRNA HCT-116 clone cells. (B) Densitometry was used to quantify relative p-β-catenin protein levels normalized to t-β-catenin protein levels. *P < 0.05 compared with NC-pEX4; #P < 0.05 compared with NC-shRNA group.

## Slide 3
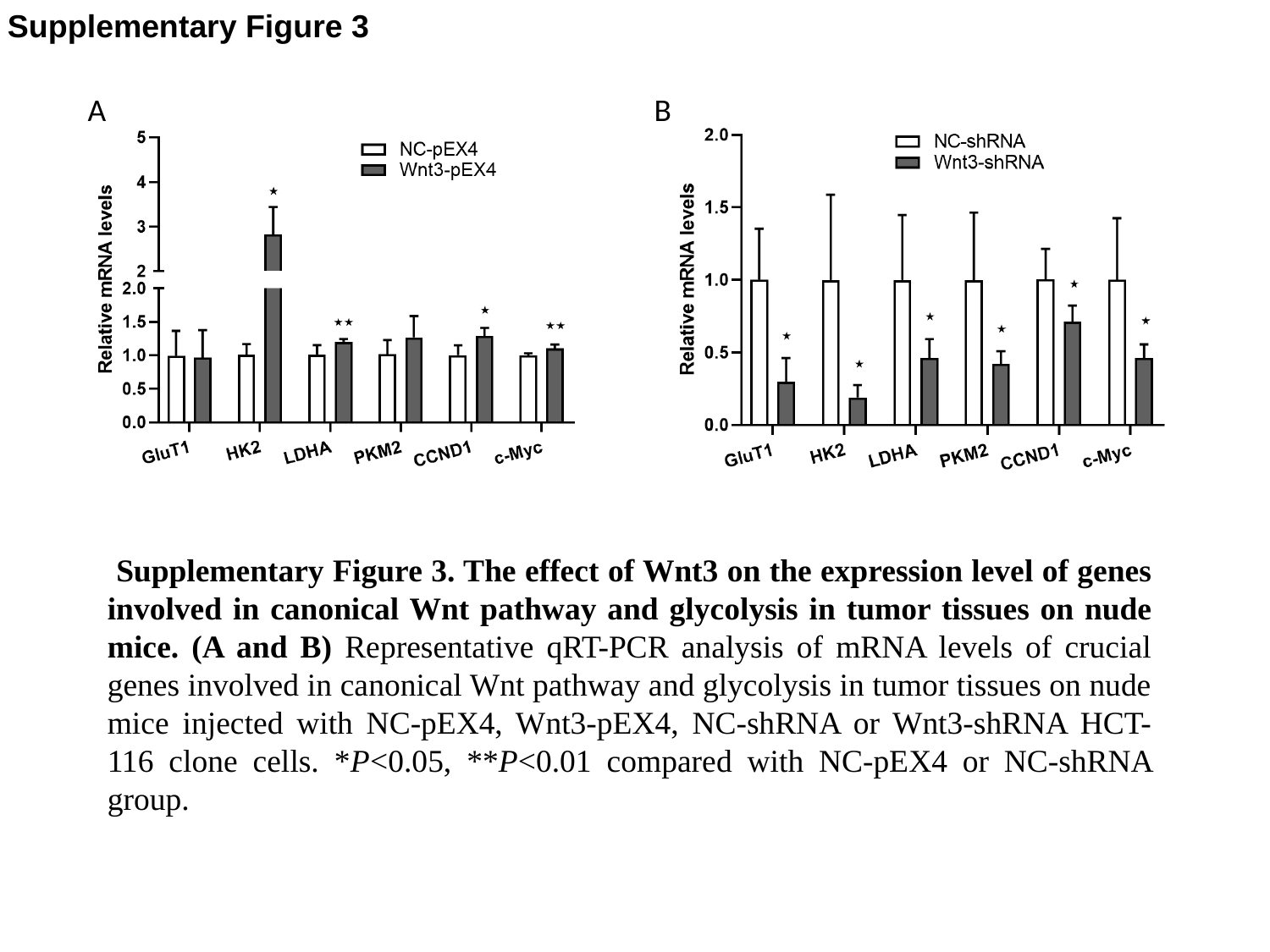

Supplementary Figure 3
A
B
 Supplementary Figure 3. The effect of Wnt3 on the expression level of genes involved in canonical Wnt pathway and glycolysis in tumor tissues on nude mice. (A and B) Representative qRT-PCR analysis of mRNA levels of crucial genes involved in canonical Wnt pathway and glycolysis in tumor tissues on nude mice injected with NC-pEX4, Wnt3-pEX4, NC-shRNA or Wnt3-shRNA HCT-116 clone cells. *P<0.05, **P<0.01 compared with NC-pEX4 or NC-shRNA group.

## Slide 4
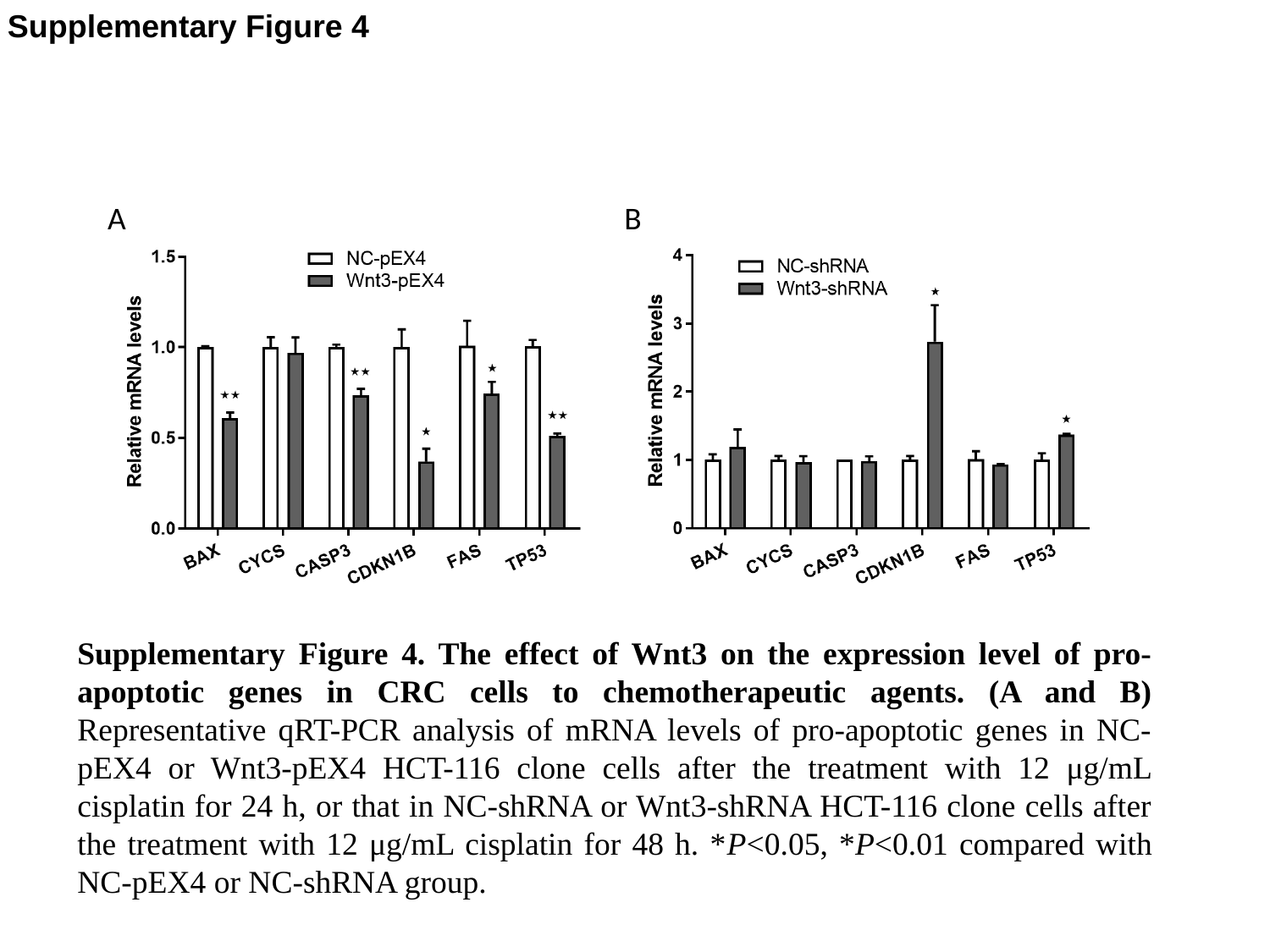

Supplementary Figure 4
A
B
Supplementary Figure 4. The effect of Wnt3 on the expression level of pro-apoptotic genes in CRC cells to chemotherapeutic agents. (A and B) Representative qRT-PCR analysis of mRNA levels of pro-apoptotic genes in NC-pEX4 or Wnt3-pEX4 HCT-116 clone cells after the treatment with 12 μg/mL cisplatin for 24 h, or that in NC-shRNA or Wnt3-shRNA HCT-116 clone cells after the treatment with 12 μg/mL cisplatin for 48 h. *P<0.05, *P<0.01 compared with NC-pEX4 or NC-shRNA group.

## Slide 5
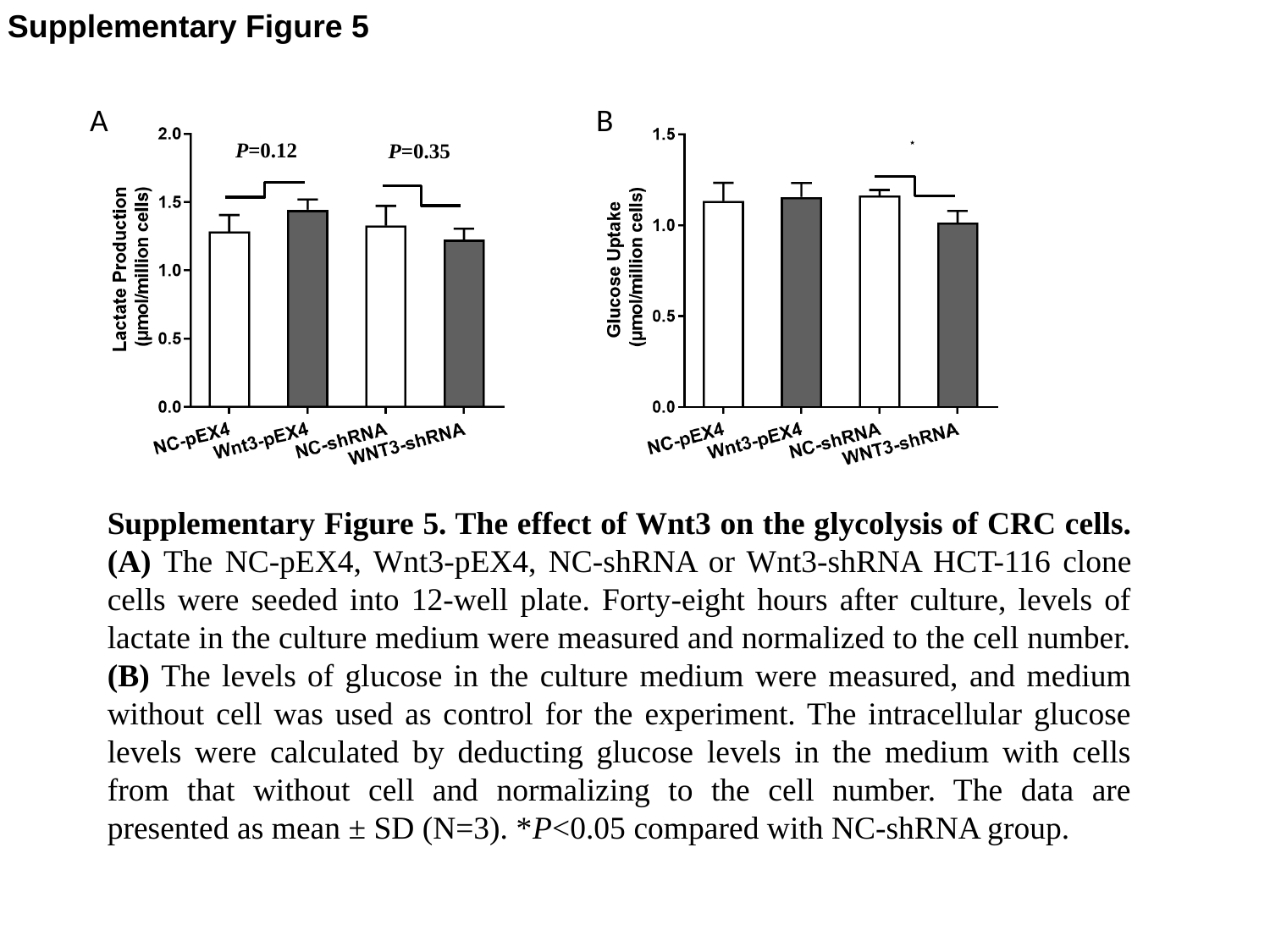

Supplementary Figure 5
A
B
★
P=0.12
P=0.35
Supplementary Figure 5. The effect of Wnt3 on the glycolysis of CRC cells. (A) The NC-pEX4, Wnt3-pEX4, NC-shRNA or Wnt3-shRNA HCT-116 clone cells were seeded into 12-well plate. Forty-eight hours after culture, levels of lactate in the culture medium were measured and normalized to the cell number. (B) The levels of glucose in the culture medium were measured, and medium without cell was used as control for the experiment. The intracellular glucose levels were calculated by deducting glucose levels in the medium with cells from that without cell and normalizing to the cell number. The data are presented as mean ± SD (N=3). *P<0.05 compared with NC-shRNA group.
